# P-1093. Role of antiseptic formulation/treatment protocols in reduction of S. aureus colonization on ex-vivo porcine tissue model

**DOI:** 10.1093/ofid/ofaf695.1288

**Published:** 2026-01-11

**Authors:** Ranjani Parthasarathy, Shazia Siddiqui, Isaac Salvatierra, Marnie Peterson

**Affiliations:** Solventum, St. Paul, Minnesota; NAMSA, Irvine, CA; NAMSA, Irvine, CA; Unaffiliated, Jackson, Wyoming

## Abstract

**Results:**

SNA showed persistent bactericidal activity ( > 5.5 log reduction compared to saline) at all time points from 1 hour to up to 5 days (1-10 treatments) with explants inoculated with MRSA (10^6^CFU/mL). In addition, SNA had a statistically significant greater log reduction compared to all other antiseptic treatments at 24 hours and 3 days and mupirocin treatment at 3 days. At 5 days, SNA was not statistically different from Nozin® and mupirocin. The mean recovery of saline for 24 hours, 3 days and 5 days was ∼5x10^7^ CFU/mL (see Figure 1).

**Conclusion:**

This study highlights the role of the antiseptic formulation/ treatment protocols for antimicrobial efficacy. Not all antiseptics provide immediate and continued antimicrobial efficacy.

**Disclosures:**

Ranjani Parthasarathy, Ph.D., Solventum: Employee|Solventum: Stocks/Bonds (Public Company) Shazia Siddiqui, MS, RM(NRCM), Solventum: Grant/Research Support Isaac Salvatierra, MB (ASCP), Solventum: Grant/Research Support Marnie Peterson, PharmD, PhD, Solventum: Advisor/Consultant

**Background:**

Recently, a study found that a nasal Iodophor antiseptic did not meet the criteria for noninferiority compared to nasal mupirocin antibiotic in reducing *Staphylococcus aureus* clinical cultures among adult ICU patients undergoing daily CHG bathing (Huang et al., JAMA, 330, no. 14 [2023]: 1337). An ex-vivo tissue model was created to test the efficacy of commercially available povidone iodine and alcohol based nasal antiseptics compared to mupirocin for one or more treatments.

**Methods:**

Porcine mucosal tissue explants (5 mm) were placed on culture inserts incubated with media and infected with methicillin-resistant *S. aureus* (MRSA) Xen30 to achieve 10⁶ CFU/explant. After 2 hour incubation, explants were treated by swabbing with antiseptic formulations (3M™ Skin and Nasal Antiseptic (SNA), Betadine® Antiseptic (10% Povidone Iodine), Profend™ and Nozin® Popswab® brands) per manufacturer’s instructions, rinsed with mucin after 1 hour and incubated for 1 hour or 24 hours at 37 °C. For the 3-day and 5-day study, explants were swabbed twice daily with antiseptic and mupirocin treatments, followed by mucin rinsing. Infected explants were collected, treated with neutralizing solution, vortexed, sonicated, and plated.

